# Multiple strategy peer-taught evidence-based medicine course in a poor resource setting

**DOI:** 10.1186/s12909-017-0924-1

**Published:** 2017-05-04

**Authors:** Ammar Sabouni, Yamama Bdaiwi, Saad L. Janoudi, Lubaba O. Namous, Tarek Turk, Mahmoud Alkhatib, Fatima Abbas, Ruba Zuhri Yafi

**Affiliations:** 1 Kasr Al-Ainy, Faculty of Medicine, Cairo University, Cairo, Egypt; 2 Faculty of Medicine, Damascus University, Fayez Mansour St. Al-Mezzeh, Damascus, Syria

**Keywords:** Evidence-based medicine, Evidence-based Health Care., Peer-taught, Medical Education., Online courses, Berlin Questionnaire., Undergraduate., Middle East., Syria., Egypt.

## Abstract

**Background:**

Teaching Evidence Based Medicine (EBM) is becoming a priority in the healthcare process. For undergraduates, it has been proved that integrating multiple strategies in teaching EBM yields better results than a single, short-duration strategy. However, there is a lack of evidence on applying EBM educational interventions in developing countries. In this study, we aim to evaluate the effectiveness of a multiple strategy peer-taught online course in improving EBM awareness and skills among medical students in two developing countries, Syria and Egypt.

**Methods:**

We conducted a prospective study with pre- and post- course assessment of 84 medical students in three universities, using the Berlin questionnaire and a set of self-reported questions which studied the students’ EBM knowledge, attitude and competencies. The educational intervention was a peer-taught online course consisting of six sessions (90 min each) presented over six weeks, and integrated with assignments, group discussions, and two workshops.

**Results:**

The mean score of pre- and post-course Berlin tests was 3.5 (95% CI: 2.94–4.06) and 5.5 (95% CI: 4.74–6.26) respectively, increasing by 2 marks (95% CI: 1.112–2.888; *p*-value <0.001), which indicates a statistically significant increase in students’ EBM knowledge and skill, similar to a previous expert-taught face to face contact course. Self-reported confidences also increased significantly. However, our course did not have a major effect on students’ attitudes toward EBM (1.9–10.8%; *p*-value: 0.12–0.99).

**Conclusion:**

In developing countries, multiple strategy peer-taught online courses may be an effective alternative to face to face expert-taught courses, especially in the short term.

**Electronic supplementary material:**

The online version of this article (doi:10.1186/s12909-017-0924-1) contains supplementary material, which is available to authorized users.

## Background

Teaching Evidence Based Medicine (EBM) to undergraduate and postgraduate medical students is becoming a priority in the healthcare process. In the UK, EBM has become a part of the foundation year program, [1] and in the US and Canada, accreditation standards for medical schools include the practice of EBM [2, 3].

In 2014, an overview systematically assessed systematic reviews published between 1993 and 2013 on teaching evidence-based medicine (EBM) in a variety of settings [4]. For undergraduates, it proved that integrating multiple strategies (lectures, tutorials, journal clubs, workshops, online courses and integrated methods) produces better results compared to a single, short duration strategy. It took into consideration outcomes such as EBM knowledge, skills, attitude, and practice.

Developing countries have less EBM awareness. In Egypt, there are wide misconceptions about EBM; most physicians consider themselves to be practicing EBM while in fact they are not [5]. In Turkey, only 1% of physicians attended EBM courses during their university life [6] and in Saudi Arabia, 13% of medical students had ever attended a course on EBM [7]. While in the United States, 38.5% of medical schools have a formal EBM curriculum [8, 9].

The barriers to EBM awareness in developing countries are several; in Iran, a systematic review was conducted to investigate obstacles to EBM, to find the most important factors which were; the absence of proper facilities, positive attitudes and adequate training [10]. In Syria, Alahdab et al. explored the barriers to EBM awareness in 2012, and reported that the most important barriers were; the absence of EBM curricula, equipment and facilities, in addition to difficulties in accessing information, institutional subscriptions to medical journals, and sufficient IT hardware [11]. In the Middle East especially, additional challenges have aggravated the situation since the Arab Spring began, such as difficulties in attending face to face contact courses due to lack of safety, resources and infrastructure. The availability of content experts is also an issue with an increase in the number of emigrating doctors; in the last couple of years in Syria alone 80,000 doctors have emigrated [12].

For all these reasons, developing countries should create their own solutions and build their experience using the available means. One possible approach is online courses, which provide flexibility in time, place, and cost [13] leading to a comparable level of knowledge gained compared with lecture-based courses [14]. Another approach is peer organised courses which cost less, need less highly experienced staff, and make it possible to generate new peers from one expert. Yet are effective in increasing self-reported confidences [15]. There is still however a lack of knowledge with regards to the effectiveness of these approaches in developing countries.

In this study, we aim to evaluate the effectiveness of a multiple strategy peer-taught online course in improving EBM awareness and skills among medical students in two developing countries, Egypt and Syria.

## Methods

### Study design

We conducted a prospective study with pre- and post- course assessment by Berlin questionnaire and a self-reported confidence questionnaire through March and April 2015.

### Participants and peers

Eighty four graduate entry medical students and recently graduated final year medical students at the Faculties of Medicine of Damascus University, Syria, Cairo University, Egypt, and Tanta University, Egypt, were enrolled in an online EBM course. Participants had little or no previous EBM skills and knowledge. The course was provided by peers using social media, Facebook and YouTube as the main course platforms. Peers were all medical students who had no previous knowledge of EBM and underwent training in the foundations of EBM by an expert, they later self-developed their EBM skills and created and presented the course.

In the study analysis we included only 48 participants who were able to take both pre and post course questionnaires in person (Table 1). All participants were asked for a verbal consent and the study was approved by the ethical committee of Faculty of Medicine, Damascus University.

**Table 1:** Basic characteristics of included participants

		(N)	(%)
Gender	Male	25	52.1
	Female	23	47.9
City	Damascus	23	47.9
	Cairo	16	33.3
	Tanta	9	18.8
Educational level	Basic science (pre-clinical) years	13	27.1
	Clinical years	35	72.9
Observed EBM	Never	33	68.8
	Once	10	20.8
	Many	5	10.4
	Regular	0	0.0
Participated in EBM	Never	36	75.0
	Once	11	22.9
	Many	0	0.0
	Regular	1	2.1

### Conduct of the educational intervention

A six-session weekly online course was provided by peers, every session included two to four videos lasting approximately 90 min in total. The videos were developed using simple means as mobile phone camera by the peers, who presented them in the participants’ native Arabic language. An evaluation quiz was mandatory at the end of each session. The evaluation included MCQs, true-false questions, open-ended questions, and journal club discussions. The quizzes’ results were not part of the analysis.

Two practical workshops were held at each venue by four of the same peers to boost the educational process, each lasted for around 3 h. In addition to discussions which were held on social media on a Facebook group.

### Course curriculum

The course curriculum was covered by a mix of online lectures and interactive workshops covering the following topics: (a) Definition of EBM and formulating clinical questions; (b) Study designs, searching the medical literature, and structure of a scientific paper; (c) Critical appraisal; (d) Analysing the results of studies, interpreting the clinical relevance and precision of the results; (e) Systematic reviews and (f) Diagnostic studies. An Additional file containing the detailed curriculum [see Additional file 1].

All online sessions, journal club discussions and practical workshops were supervised and conducted by peers.

### Outcome assessment (the questionnaire)

The Berlin Questionnaire [16] was chosen for appraisal of the participants due to its proven validity, internal consistency, and ability to accommodate for change. It is also able to distinguish participants at different stages of the learning process [17]. The Berlin Questionnaire consists of 15 MCQ-item questions with one point for each question. Questions are based on clinical cases and appraise the learner’s ability to formulate a question, process the suitable evidence, and recognise study design. The questionnaire has a focus on therapeutic and diagnostic studies (4 questions each), and systematic review, prognostic, and harm studies (1 question each) and the rest of the question cover a number of different subjects. The complete score was 15 marks. The Questionnaire was supplemented with a set of self-reported questions to inspect students’ self-reported confidences [18]. Participants completed the pre and post-course survey provided before the first session and after the last session.

### Statistical analysis

Data were entered into a Microsoft Excel spreadsheet for each city, combined, and then imported into SPSS version 22.0 to perform the analysis.

## Results

A total of 84 students were enrolled in the course, while only 48 students were able to take both pre and post course questionnaires in person and were included in the final analysis with a response rate of 57.1%.

(Table 1) describes the basic characteristics of included participants.

### Objective and subjective evaluation of EBM skills and knowledge before and after the course

**Fig. 1:**
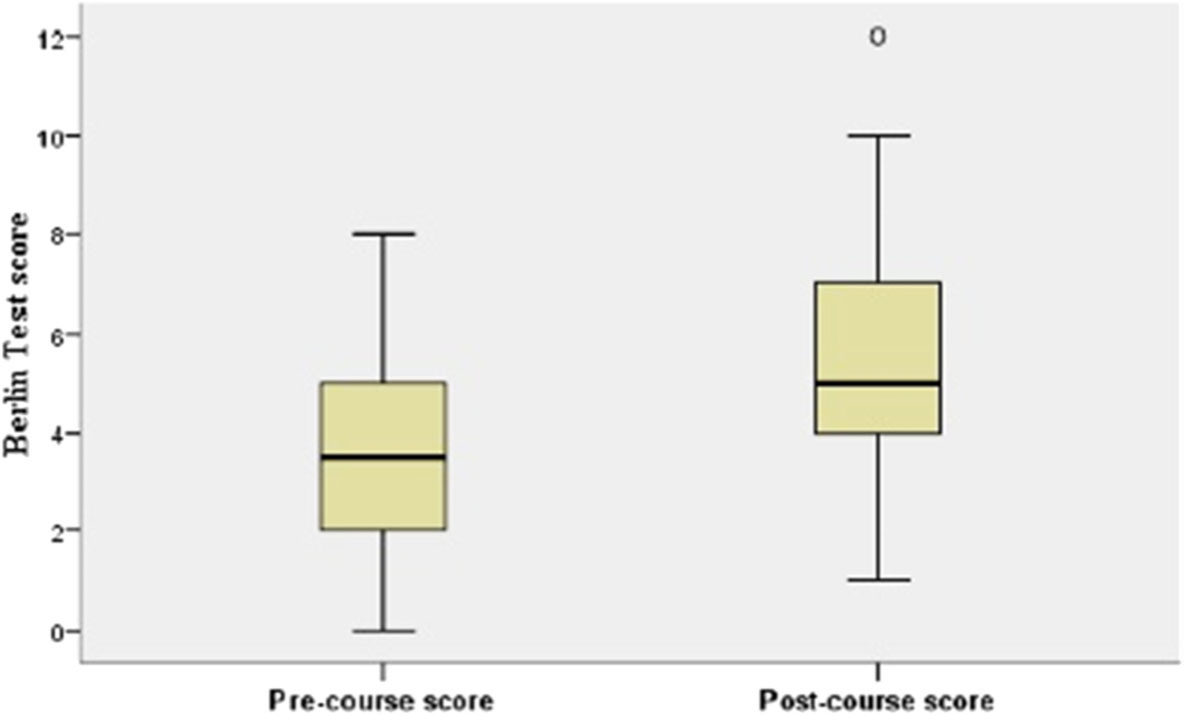
Pre- and post-test scores for participants

The mean scores of students’ pre- and post-course Berlin tests were 3.5 (95% CI: 2.94–4.06) and 5.5 (95% CI: 4.74–6.26) respectively, increasing significantly by 2 marks (95% CI: 1.112–2.888; *p*-value <0.001), (Fig. 1). Further comparisons were made to demonstrate the change between pre- and post-course test’s mean scores among different baseline factors. Females gained significantly more knowledge and skills than males. Comparing residence, education level and the current course’s impact with a previously held face-to-face course did not yield significant results (Table 2).

**Table 2:** Post-course scores’ mean difference comparing gender, residence, year of study, and current course vs. a previously held course

Variables	Post-course mean scores	Post-course mean difference
Males vs. Females	Males = 1.16	1.75 (*P*-value = 0.03)
	Females = 2.91	
Syria vs. Egypt	Syria = 2.52	1 (*P*-value = 0.25)
	Egypt = 1.52	
Basic years vs. Clinical years	Basic years = 2.07	0.105 (*P*-value = 0.69)
	Clinical years = 1.9	
Current course vs. previously held course	Alahdab et al. course = 2.65	0.65 (*P*-value = 0.14)
	Current course = 2	

### Perception and self-reported knowledge of EBM

As a self-rating of EBM skills and knowledge, all of the included participants (100%) thought they knew little or nothing at all about evidence-based medicine before the course. This percentage decreased by half (*p*-value <0.001) after completing the course.

**Fig. 2:**
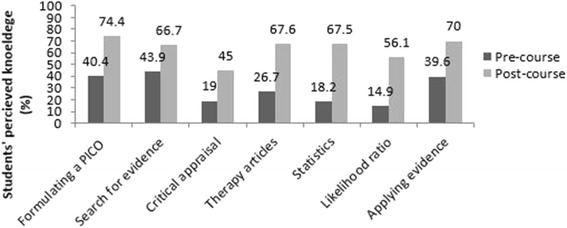
Students’ self reported confidence level of certain aspects of EBM before and after the course

Regarding self-rating of different EBM skills, 40.4% of students reported being confident/extremely confident in their ability to formulate a PICO question. This percentage increased to 74.4% after completing the course (*p*-value < 0.01). Students’ ability to perform an online literature search, calculating basic statistics, and their ability in applying evidence for patient-centred care increased significantly between the pre- and post- test (22.8% *p*-value = 0.016; 49.3% *p*-value <0.001; 30.4% *p*-value = 0.001 respectively). On investigating students’ critical appraisal skills, we found that the increase by 26% was not statistically significant (*p*-value = 0.05). Figure 2 emphasizes the key findings and differences between pre- and post-course self-reported confidences.

### Students’ attitudes toward EBM before and after the course

Table 3 summarizes all results related to students’ attitudes toward EBM before and after the course. When asked if evidence-based medicine is time-consuming, 71.7% of the students disagreed with this statement before the course, compared to 69.8% disagreeing with it after the course, this difference in attitude is not statistically significant (*p*-value >0.05). When students were asked whether EBM relies too much on statistics, 8.7% of them disagreed before the test, and this attitude was did not essentially change, as 19.5% of them disagreed with this statement after the course, a non-statistically significant result (*p*-value >0.05).

**Table 3:** Students’ attitudes toward EBM before and after the course

Variables	Pre-course percentage of students disagreeing (%)	Post-course percentage of students disagreeing (%)	Percentages difference (%)	*P*-value
EBM is time consuming	71.7	69.8	1.9	0.99
EBM is a “cookbook” for medicine	45.7	50	4.3	0.99
EBM relies too much on statistics	8.7	19.5	10.8	0.12

## Discussion

We found our multiple strategy peer-taught online course significantly improved medical student EBM skills. Participants improved with a mean increase in the Berlin questionnaire score of 2 marks. Yielding an almost identical increase in EBM knowledge to expert-taught face to face courses [11, 15, 19].

*After the course, participants’ self-confidence improved in formulating a PICO research question, performing an online literature search, reading and assessing the results of therapy-related articles, using basic statistical concepts, using likelihood ratio, and applying evidence for patient-centred care.*

### Multiple strategy course

The strategy we adopted, a multiple strategy online course presented over several weeks, accompanied with several face to face contact workshops, group discussions and assignments, proved effective in line with the findings of Young et al. Young et al. in their overview of systematic reviews suggested that integrating multiple strategies presented over a few weeks, is better than a single strategy method [4]. The results of our social media strategy which was based on Facebook and Youtube were also in line with previous findings [20].

### Online platform

Our online course was effective as a comparable alternative that saved on effort, time, and cost. Hadley et al.’s trial also reported no difference between an e-learning EBM course and a lecture-based EBM [14]. Even though we did not carry out a full cost analysis, our course only required the cost of reserving a hall for the two workshops. This corresponds with what Maloney et al. revealed in that a blended e-learning approach cost 24% less than face to face learning [13]. Online courses also serve as a reference which can be used when repeating workshops.

In addition, we expect the fact that our online course was conducted in the participant’s native language (Arabic in our case) enhanced the reach of the course to students of all capabilities and so promoted more students to join.

### Peer-taught strategy

Another approach we examined was the peer-taught strategy; we found peer teaching effective in improving students’ EBM knowledge and skill. This follows the results of a peer-taught EBM workshop held at The UK National Institute for Health and Care Excellence (NICE) [15]. We also proved efficacy similar to a two day face to face expert-taught course, previously held in Syria by Al-Ahdab et al. The difference between the two courses was non-statistically significant [11].

### Perspective

Many studies have addressed teaching EBM skills and knowledge to medical students and health professionals, and changing attitudes towards EBM in both developed and developing countries [10, 11]. In light of recent lack of safety, and lack of both human and material resources in the Middle East and North Africa region, more flexible and affordable means are necessary. Online and peer taught courses are two possible solutions yet there is limited literature on their effectiveness in teaching EBM in developing countries. Our study addressed this. Studies on effectiveness of EBM courses in general in Syria and Egypt are also scarce and may represent another unique situation in the security situation in countries in crisis and teaching in them. This study also compares between these countries.

### Limitations

Our course did not have a significant effect on students’ attitude toward EBM this may be explained by our questions not comprehensively assessing EBM attitude. In addition, our course did not significantly affect the self-confidence of students to critically appraise a study, even though there was a specific focus on critical appraisal, and was addressed in several lectures, workshops and assignments. These finding may be explained by students’ overestimation of their critical appraisal skills pre-test before delving into the details of the course.

We suggest the main limitation of our study is the subjectivity of self-reported attitudes. We cannot judge whether this improvement will be implemented in practice or not. In addition, though students reported no previous background in EBM, students voluntarily participated in the course and this may have imposed a positive bias. We think our sample size and diminished questionnaire response are also limitations. Another important limitation is that the study design did not allow for a live comparison between a face to face and an online course, a controlled trial is the better alternative.

## Conclusion

According to our findings, we suggest that to build reliable Evidence-Based Medicine practitioners in developing countries, multiple strategy peer-taught online courses are an effective approach and a comparable alternative to face to face expert-taught courses.

We recommend university role models and EBM experts implement and further assess this choice to provide high quality online courses and higher EBM awareness among medical students in developing countries.

## Additional file

Additional file 1: The course’s curriculum. (DOCX 13 kb)
