# Supplementary material for: Multiple strategy peer-taught evidence-based medicine course in a poor resource setting
Source: BMC Med Educ. 2017 May 4;17:82. doi: 10.1186/s12909-017-0924-1 (PMC5418757; doi:10.1186/s12909-017-0924-1)
Supplement: Additional file 1: — The course’s curriculum. (DOCX 13 kb) [file 12909_2017_924_MOESM1_ESM.docx]

#### **Course Curriculum**

In the first session, we introduced participants to the concept of EBM and how it is used in clinical practice. They were taught to recognize situations that involve clinical uncertainty and how to frame a research question in the PICO structure (patient-intervention/exposure-comparator-outcome). Expressing clinical questions through PICO involves asking a comprehensive research question that clearly addresses the population, interventions, comparisons and outcomes related to the investigated topic.

The second session was dedicated to understanding the importance of each type of study design, to choose the best study design for a specific clinical question, learn the advantages and disadvantages of each type, and the hierarchy of evidence. Also covered were online databases, search engines, and practically searching the medical literature for a published paper. Due to the limited internet access and online resources at the universities and their affiliated hospitals, participants only practiced on MEDLINE PubMed. Finally, they learned the structure of a scientific paper, the distinct sections of a paper and the type of information each section contains.

The third session covered critical appraisal of evidence for validity, clinical relevance, and applicability. The students were taught to appraise the validity of a randomized clinical trial. The appraisal included: the suitability of the type of study to the type of question asked, the design of the study and sources of bias, the reliability and validity of outcome measures chosen, and the suitability and robustness of the analysis employed.

In the fourth session, students learned to appraise the importance of the outcomes of a scientific paper and to translate them into clinically meaningful statistical concepts, such as number needed to treat (NNT), absolute risk reduction (ARR) and relative risk reduction (RRR), and interpretation of confidence intervals (CI).

The fifth session included an introduction and explanation of systematic reviews and meta-analyses, and also covered steps to perform a systematic review. We then presented a critical appraisal exercise using predetermined criteria and an analysis of how to interpret different types of evidence.

In the last session, participants were taught measures of diagnostic accuracy including: sensitivity, specificity, positive and negative predictive value, and likelihood ratios.

This content was also presented as an online journal club and two live workshops. In the journal club, participants were provided with a study to read and critically appraise, the critical appraisal was later explained in an online session, and discussed on the Facebook group. The live workshops contained welcoming instructions and an introduction into EBM in the agenda of the first. In the second there was a questions and answer session, followed by an interactive discussion between the participant and the facilitator peers on the course content.
